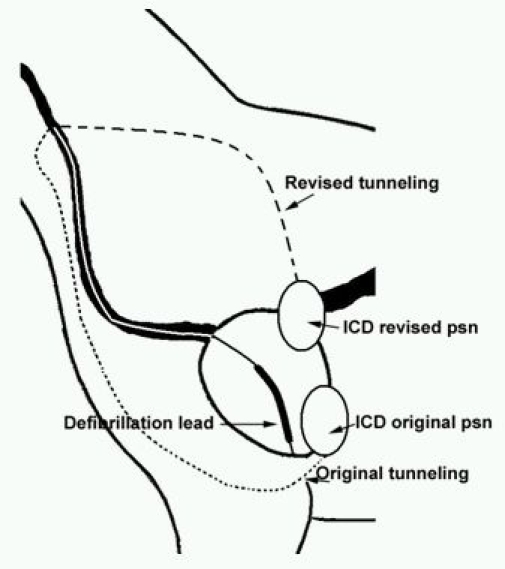# "Twiddler" In A Canine Model And A Modified Implant Technique To Prevent It

**Published:** 2009-05-15

**Authors:** Sony Jacob, Prasad K Cherian, Elizabeth Dawe

**Affiliations:** Cardiac Experimental Electrophysiology Laboratory, Wayne State University, Detroit, MI, USA

**Keywords:** Twiddler's Syndrome, Canine Model, ICD

This report explains the Twiddler's syndrome, a well known human entity, in a canine model. We also describe a modified technique to implant internal cardioverter defibrillators (ICD) in dogs to prevent Twiddler's syndrome. Our original study was to identify the factors influencing the DFT measurement and a canine model was selected as it is the best proven electrophysiological model. During the course of the study, one of the dogs was evaluated for device failure at three weeks following implantation. While under general anesthesia, interrogation of the dog's ICD showed that the right ventricular (RV) pacing threshold was high and ventricular capture was intermittent. When examined under fluoroscopy, it was noted that the defibrillator lead was dislodged from the right ventricular apex. A surgical revision of the device pocket showed that the leads were twisted in an almost braided fashion - as if "twiddled" into position around the device [Figure 1 (arrow)]. We suspect that this "twiddling" resulted in shortening and pulling of the lead from the ventricular myocardium causing intermittent capture and device failure.

Twiddler's syndrome, a not uncommon occurrence in human clinical cardiology practice, was first described by Bayliss et al [1]. It is likely to occur when an implanted pulse generator or ICD is rotated in the subcutaneous pocket resulting in malfunction of the device. Interestingly, Twiddler's syndrome has also been reported in animals [2]. Twiddling of the pacemaker leading to twisting of the lead in animals may be more common than reported as it is usually implanted in a pocket fashioned in the neck region where the subcutaneous tissue is loose. In our dog, during the original implantation, the RV lead was implanted via the left external jugular vein (LEJ) access [Figure 1 (arrowhead)] and the lead was tunneled deep to the subcutaneous tissue via the left ventrolateral neck over the distal end of the left humerus to a subcutaneous pocket prepared by digital dissection on the left lateral thorax. The "twiddling" may be caused by scratching of the cranial chest region with a hind paw or may be due to local muscular action during normal activities [2]. A surgical revision was performed in our dog where the "twiddled" lead was untwisted, extracted and a new defibrillator lead was implanted and secured in RV apical position. To prevent the "twiddling", the new lead was tunneled deep to the subcutaneous tissue over the dog's left shoulder dorsally from the LEJ to a new subcutaneous device pocket in the lateral chest wall. (Figure 2) This approach essentially minimized the device movement and hence "twiddling" by the hind paw. We observed the dog closely for three months post re-implantation; the sensing and pacing threshold and lead impedance were within normal limits. Since animal models are being used more frequently to perform electrophysiological studies, this observation of Twiddler's Syndrome in the canine may be of interest.

The entire study was done under general anesthesia with special emphasis on perioperative and postoperative pain management. All experimental procedures and protocols used in this investigation were reviewed and approved by the Animal Care and Use Committee of the Wayne State University. All conformed to the "Guiding Principles in the Care and Use of Animals" of the American Physiological Society and the Guide for the Care and Use of Laboratory Animals of the National Institutes of Health (Revised, 1996).

## Figures and Tables

**Figure 1:**
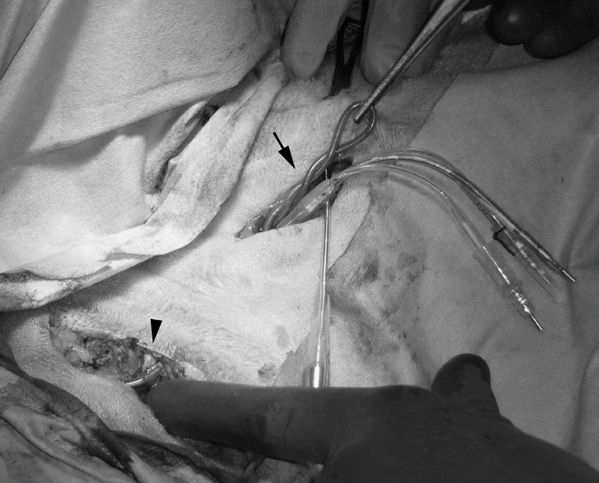


**Figure 2:**